# Pancreatic Cancer and Its Microenvironment—Recent Advances and Current Controversies

**DOI:** 10.3390/ijms21093218

**Published:** 2020-05-01

**Authors:** Kinga B. Stopa, Agnieszka A. Kusiak, Mateusz D. Szopa, Pawel E. Ferdek, Monika A. Jakubowska

**Affiliations:** 1 Malopolska Centre of Biotechnology, Jagiellonian University, ul. Gronostajowa 7A, 30-387 Krakow, Poland; kinga.stopa@doctoral.uj.edu.pl; 2 Faculty of Biochemistry, Biophysics and Biotechnology, Jagiellonian University, ul. Gronostajowa 7, 30-387 Krakow, Poland; a.kusiak@student.uj.edu.pl (A.A.K.); mateusz.szopa@student.uj.edu.pl (M.D.S.)

**Keywords:** CAFs, microenvironment, pancreatic cancer, PDAC, signaling, stroma

## Abstract

Pancreatic ductal adenocarcinoma (PDAC) causes annually well over 400,000 deaths world-wide and remains one of the major unresolved health problems. This exocrine pancreatic cancer originates from the mutated epithelial cells: acinar and ductal cells. However, the epithelia-derived cancer component forms only a relatively small fraction of the tumor mass. The majority of the tumor consists of acellular fibrous stroma and diverse populations of the non-neoplastic cancer-associated cells. Importantly, the tumor microenvironment is maintained by dynamic cell-cell and cell-matrix interactions. In this article, we aim to review the most common drivers of PDAC. Then we summarize the current knowledge on PDAC microenvironment, particularly in relation to pancreatic cancer therapy. The focus is placed on the acellular stroma as well as cell populations that inhabit the matrix. We also describe the altered metabolism of PDAC and characterize cellular signaling in this cancer.

## 1. Introduction

Approximately 95% of the pancreas is composed of the highly specialized epithelial cells [1]. Secretory epithelia, pancreatic acinar cells (PACs) and pancreatic duct cells (PDCs), are characterized by highly specialized functions, reflected by their sophisticated cell architecture and structural polarity [2]: apico-basal for the acinar cells [3] and planar for the duct cells [4]. It is now commonly accepted that in the normal epithelial tissues, the maintenance of cell polarity is fundamental for preservation of tissue integrity and functions [5]. However, in the process of malignant tissue transformation, this physio-functional propensity of the epithelia becomes severely disrupted [5].

Physiologically, pancreatic secretory epithelia play unique roles in the digestion. The main role of PACs is production, storage (as zymogen granules) and release of digestive enzymes [6], whereas PDCs secrete water and electrolyte (HCO_3_^−^), which, together with the enzymes, formulate alkaline pancreatic juice [7].

Although seemingly ‘terminally’ differentiated, PACs and PDCs have the ability to reversibly assume the phenotype characteristic of other cells. For instance, in response to the injury, acinar cells may become transdifferentiated into duct-like cells and develop acinar-to-ductal metaplasia (ADM); and vice versa: ductal-to-acinar (DAM). In each case, one predominant cell type becomes replaced by another [8,9,10]. ADM is characterized by remarkable cell plasticity, often apparent under cellular stress (e.g., recurrent inflammation), whereby the presence of oncogene *KRAS* may further cell transformation into benign stages called pancreatic intraepithelial neoplasia (PanINs) [9,10,11,12]. By contrast, withdrawal of the stressor may favor a reversion toward the primary acinar phenotype [9,13]. However, as a result of the secondary genetic aberrations (e.g., inactivation of tumor-suppressor genes), PanINs may undergo a malignant transformation, which leads to the development of pancreatic ductal adenocarcinoma (PDAC) [9].

Importantly, even at early PanIN stages, pancreatic epithelia may transdifferentiate toward the mesenchymal fate in the program(s) called epithelial-mesenchymal transition (EMT) [14]. This transition causes marked changes of the phenotype: not only manifested as the loss of polarity, but also as disruption of the contact-mediated (cell-cell and cell-matrix) signaling [5,9,14]. What is more, a neoplastic cell increases its potential for migration and may leave the original niche and enter the bloodstream. In circulation, the mesenchymal phenotype is maintained until the cell has reached a new habitable niche [14]. If the cell was malignantly transformed, this process can initiate metastasis formation [14]. However, it remains controversial whether or not the presence of pancreatic epithelial cells in circulation would deliver any relevant diagnostic/prognostic data for the patients [14,15,16].

The abundance and complexity of the precursor stages, the ease to disseminate into distant metastatic niches and the ability to undergo EMT, all make PDAC particularly difficult to cure. The statistical data of the American Cancer Society website: *cancer.org*, convey a tragic message that the 5-year relative survival rate post diagnosis, combined for all SEER stages (Surveillance, Epidemiology, and End Results), is about 9%. In this review article, we aim to summarize the mutations that initiate PDAC development. We then focus on the specific PDAC microenvironment formed by the acellular matrix and populations of tumor-associated cells. Finally, we characterize the role of hypoxia, altered cancer metabolism and microbial flora, as well as fat and calcium signaling pathways in the pancreatic carcinogenesis.

## 2. Driver Mutations in PDAC

Pancreatic carcinogenesis is largely driven by mutations in certain oncogenes and tumor suppressor genes. This includes activation of the *KRAS* oncogene as well as inactivation of *CDKN2A* tumor suppressor gene, followed by a loss of function of *p53* and *SMAD4* [17]. Other less frequent contributors are mutations in *DPC4*, *PTEN*, *Myc* and *BRCA2* genes [18,19]. In 2008 the pioneering work by *Jones* et al. applied microarrays containing probes for ~1 million single-nucleotide polymorphisms to identify somatic mutations in 24 pancreatic cancer samples [20]. This study described well over 1500 somatic mutations, majority of which were missense (62.4%) and synonymous (25.5%) mutations [20].

### 2.1. Activation of Oncogenes—KRAS

In human cancers, *KRAS* mutation is considered to be one of the most common driver mutation in the lung, colorectal and pancreatic carcinogeneses [21]. Activation of the *KRAS* oncogene occurs early in the course of PDAC progression and it can account for up to 30% of the non-invasive ductal lesions, increasing to almost 95% in aggressive PDAC [22,23].

KRAS is an intracellular membrane-bound protein that belongs to the small GTPase superfamily, involved in regulation of the cell cycle, cell proliferation, differentiation, metabolism and apoptosis [24,25]. KRAS can exist in two different states: the inactive GDP-bound and the active GTP-bound, which enables it to act as a molecular switch [23]. As a consequence of the point mutations in codon 12 (also 13 or 61) resulting in amino acid substitution (e.g., KRAS^G12D^, KRAS^G12C^ [26]), KRAS becomes resistant to inactivation by GTPase activating proteins (GAPs) [19]. This in turn leads to the constitutive KRAS activity and a subsequent overstimulation of cell proliferation and pro-survival signaling, including PI3K and RAF/MAP/MEK pathways [17]. Activated KRAS is also involved in a number of signaling cascades that regulate cell metabolism, reprogramming, as well as inflammation and angiogenesis [25]. What makes the matters worse is that these genetic alterations occur not only in cancer cells, but may also be present in the tumor microenvironment, for example in pancreatic stellate cells (PSCs) [27].

In the scientific community it is commonly perceived that an effective therapy that could selectively target KRAS might become an important step forward in the PDAC treatment. Currently developed therapeutic approaches address this concept, and some indirect strategies attempt to affect cellular location of KRAS itself or target its downstream effectors [25,28,29].

One of the first therapeutic approaches proposed to decrease KRAS activity was inhibition of mutated *KRAS* expression. This was attempted by application of a small interfering RNA (siRNA) called ISI6957 [30]. However, due to the short circulating half-life, inefficient cellular uptake and off-target toxicity (that is toward normal cells), this therapy did not proceed into the clinic [30,31]. Only very recently, peptide-based nanoparticles have been validated as safe and effective carriers of siRNA to the target cells; this strategy was successfully applied to inhibit *KRAS* expression, which resulted in reduced viability of cancer cells, as tested in vitro and in a KPPC mouse model of PDAC [29].

Another tested therapeutic approach was based on irreversible inhibition of KRAS by a disruption of the conformational changes required for oncoprotein activation. Although conceptually attractive, in practice high intracellular levels of GTP as well as significant affinity of KRAS toward this nucleotide were able to overcome the induced conformational inhibition resulting in the failure of this strategy [25]. Currently, under clinical trials is the first in its class compound AMG 510 that covalently binds to the extra cysteine residue in KRAS^G12C^ and abolishes the activation of mutated KRAS by locking this protein in its inactive state [32,33].

Recently, increasingly more attention is directed toward post-translational modifications of KRAS, particularly those that inhibit docking of the oncoprotein to the cell membrane and thus affect its intracellular localization. While some of the simplest modifications include prenylation and geranylgeranylation of KRAS, their translation into the clinic is rather unlikely due to the predicted high toxicity [34]. A promising yet unproven approach to target KRAS docking to the plasma membrane could be the application of Deltarasin - an inhibitor of the prenyl-binding chaperon protein PDEδ [35,36]. Since the efficacy of Deltarasin has so far been demonstrated only in the preclinical models, clinical research is still required to test the drug in the human patients [35,36].

Several compounds have been developed to block the pathways downstream of KRAS. Those include RAF and MEK inhibitors disrupting the MAPK signaling pathway. However, studies failed to demonstrate efficacy in PDAC treatment for Selumetinib and Pimasertib, blockers of MEK the MAPK kinase [37,38], or Rigosertib - historically known as a RAS mimetic [39], and recently identified as a microtubule destabilizing agent [25,40,41,42,43].

In 2003, a mouse model of PDAC was first used to demonstrate that mutation in KRAS is an initiating event in pancreatic ductal carcinogenesis [18]. In these mice, expression of endogenous KRAS^G12D^ in progenitor cells was found to be sufficient for the induction of benign lesions, such as PanINs the initial stage of pancreatic carcinogenesis [18,25]. What might be surprising is the fact that there is only 1% chance that a KRAS mutation-carrying PanIN1 will progress to PDAC [44,45]. However, an effect of mutated KRAS in PDAC could be intensified in the pre-malignant lesions by the presence of the secondary mutations that promote the development of aggressive disease. Indeed, a recent study on transcriptional regulator ATRX (involved in chromatin remodeling) showed that the loss of this protein in female but not male mice increases the susceptibility to pancreatic injury and oncogenic KRAS [45]. These findings point toward the existence of additional (epi)genetic changes that are essential for pancreatic carcinogenesis.

### 2.2. Inactivation of Tumor Suppressor Genes

#### 2.2.1. CDKN2A

Loss of function of cyclin-dependent kinase inhibitor 2A (CDKN2A; also known as p16) occurs in more than 85% of PDAC cases [19,46,47,48,49]. The *CDKN2A* gene is frequently exposed to intragenic mutations, homozygous deletions and promoter hypermethylation, all of which result in a non-functional protein [50]. Frequency of p16 inactivation increases during pancreatic carcinogenesis, and can be identified at its very early stages [51]. Under normal conditions, p16 acts as a regulator of the cell cycle and inhibitor of cyclin-dependent kinases 4 and 6 (CDK4, CDK6) activation. Blockage of these kinases disables their interaction with cyclin D1, phosphorylation of retinoblastoma protein (Rb) and halts progression of the G1/S phase cell cycle [50]. Under pathological conditions, loss of functional p16 leads to the unsuppressed cell proliferation, often resulting in PDAC development [52]. Currently ongoing clinical trials (NCT03065062, NCT02501902) test inhibition of CDK4 and CDK6 by Palbociclib, in combination therapies against PDAC.

#### 2.2.2. TP53

In 75% of the PDAC patients, the *TP53* suppressor gene is either completely lost or carries missense mutations [53,54]. Mutations in this gene are present in advanced PanIN-3 and invasive cancers, and are often considered to be late events in pancreatic carcinogenesis [51]. *TP53* encodes a transcription factor p53, also called The Guardian of the Genome, since it coordinates the whole cell genome function by regulating the cell cycle, metabolism and apoptosis in response to DNA damage [17,19]. It is not surprising that in normal cells the level of p53 is under precise control. In these cells, p53 is targeted for proteasomal degradation by E3 ubiquitin-protein ligase MDM2. This post-translational control of p53 level and its activity leads to the rapid response that enables the cell cycle arrest as a result of DNA damage. In cancer, p53 mutant protein interacts with the unchanged p53 protein blocking its normal functions. Despite the above, the effect of *TP53* mutations alone on carcinogenesis initiation is rather minor. It is the simultaneous occurrence of mutations in *TP53* and *KRAS* that usually leads to the development of metastatic carcinoma [55].

While not essential for the initiation of pancreatic malignancy, *TP53* mutations are responsible for a decreased chemotherapeutic efficiency of PDAC. As a result, various approaches that aim to deprive *TP53* or restore its wild-type activity are within scientific and medical interest. One of the emerging therapeutic strategies currently under (pre)clinical evaluations is based on inhibitors of heat shock protein 90 (HSP90). The chaperone activity of HSP90 is important for the transcriptional activity of wild-type p53 [56]; and thus blocking of HSP90 activity could destabilize p53 [57,58]. Also, inhibitors of histone deacetylases (HDAC), e.g., Trichostatin A, which bind directly to the *TP53* gene, can reduce expression of this gene and may prove beneficial in PDAC treatment, subject to further validation in the clinic [59].

#### 2.2.3. SMAD4

Mutations in *SMAD4* have been reported in approximately 60% of PDAC cases [60]. SMAD4 is a regulator of transcription growth factor-beta (TGFβ) signaling pathway that controls cell proliferation, apoptosis, migration and EMT [61]. The loss-of-function mutation in *SMAD4* is typical for the advanced stages of PDAC and is correlated with metastasis formation [62]. This makes *SMAD4* expression a prognostic factor for the PDAC progression or assessment of the therapy outcome [62].

Importantly, the above-listed mutations do not occur exclusively in cancer cells. It was shown that inactivation of *TP53* and *PTEN* can also be found in the stromal cells where it promotes the progression of different types of cancers [63,64,65].

### 2.3. Non-Coding RNAs

Apart from the driver mutations, dysregulation of non-coding RNAs (ncRNAs) was shown to influence PDAC initiation and progression. ncRNAs comprise microRNAs (miRNAs), the endogenous single-stranded RNAs (ssRNAs) and long non-coding RNAs (lncRNAs). miRNAs specifically target mRNA for degradation or translation repression; they may also act as decoys for certain proteins [66,67]. The role of lncRNAs is more heterogeneous: these ncRNAs can influence the transcription itself as well as post-transcriptional processes and chromatin remodeling [67].

In order to regulate vital cellular processes, e.g., cell growth and differentiation, angiogenesis, migration and apoptosis, miRNAs and lncRNAs are present both in normal and diseased tissues [68,69]. However, in pathology the expression levels of ncRNAs may not change sharply enough to be used as early diagnosis/prognostic markers or the potential targets in anticancer therapies [68,70].

miRNAs found in PDAC can be divided into two groups: *onco*miRs (miR-21, miR-155 and miR-221) and *ts*miRs (miR-146, miR-34 and let-7), based on their oncogenic or tumor suppressing role, respectively [69]. For example, miR-21 expression in PanINs corelates with PDAC aggressiveness, possibly owing to its regulatory role in PI3K signaling, mobilizing CAFs and increasing cancer resistance to therapies [69,71]. It was recently shown that downregulation of *onco*miR-21 significantly slows down the kinetics of tumor growth in vivo [72]. As for the *ts*miRs, downregulated in PDAC tumor suppressor let-7 was demonstrated to modulate cell proliferation and EMT and thus promote pancreatic tumorigenesis [73]. Restoration of let-7b level in combination with GDC-0449 (inhibitor of the Hedgehog signaling) reduced growth of MIA PaCa-2 tumors in nude mouse hosts [74].

Despite the importance of ncRNAs in carcinogenesis, oligonucleotides did not proceed to the clinical trials for PDAC treatment. One of the reasons is a very short in vivo half-life of ncRNAs due to the activity of serum nucleases and renal elimination [75]. However, current preclinical studies aim to target this disadvantageous pharmacokinetics of oligonucleotides in order to increase the efficacy of this therapy, e.g., chemically tailored nanocomplexes have been tested as vehicles for delivery of anti-miRNA oligonucleotides (AMOs). These nanocomplexes demonstrated a remarkable success in trafficking AMOs to the PSCs, wherein they blocked specifically miR-199a and subsequently constrained PSC activation [75].

## 3. Microenvironment: Acellular Stroma

Comprising of ~80% of the tumor microenvironment (TME), the stroma is an important part of PDAC [76], that functionally can be divided into four subclasses: (i) structural vascularized, (ii) activated, (iii) inflammatory, and (iv) immune [77]. In order to perform its structural and connective roles, the stroma must contain both the acellular matrix formed mainly of water and glycoproteins, and live components non-neoplastic cells. Cellular composition of pancreatic cancer milieu displays a remarkable diversity of cell populations, including fibroblasts, endothelial cells, immune cells (lymphoid and myeloid cells) and even neurons. A predominant fraction of the PDAC stroma are PSCs, that have assumed an active myofibroblast-like phenotype in TME [78]. Dynamic interactions of these cells with other tumor components shape the extracellular matrix (ECM) and influence the neoplasm progression [79].

ECM forms the ‘ecosystem’ of physical/mechanical cues and makes possible interactions between the structural proteins and other matrix components that are essential for the preservation of tissue integrity. Dynamic structure of ECM is upheld by a constantly changing ratio of matrix metalloproteinases (MMPs) and their inhibitors TIMPs, which is influenced by IL-1α and TGFβ signalings [80]. These cellular signaling pathways remodel/reorganize ECM both in cancer and normal tissue, further enhancing it shielding properties [81].

Collagens (COLs) are the major components of ECM. In PDAC, collagens play an intricate role: they reinforce the tumor composition by interacting with α_2_β_1_ integrins that regulate fibril motility and fibril binding to a number of proteins [82]. COL1A influences E-cadherin level and indirectly mediates formation of β-catenin/E-cadherin complexes that facilitate PDAC progression [83]. Moreover, collagen I promotes the metastatic potential of PDAC by upregulation of N-cadherins by Discoidin domain receptors (DDRs) [84]. PDAC growth can also be affected by the loss of the *PTEN* gene that encodes phosphatase and tensin homolog (PTEN) protein: this causes higher mobilization of CAFs and eventually a more efficient production of collagen type I [85,86]. What is worth noticing is that not only the presence but also the spatial organization of collagen is important for cancer development. As opposed to chronic pancreatitis and the healthy pancreas, collagen fibers in PDAC exhibit a characteristic topology, particularly around malignant ducts, determined by a high alignment and increased length and width [87]. Such a distinctive organization of collagens in the tumor often implies a poor prognosis for patient survival [88].

In research focused on PDAC stroma, polysaccharide hyaluronan (HA) emerges as an extensively studied component of the acellular matrix. Importantly, the abundance of HA in the stroma was linked with improved angiogenesis in the tumor parenchyma, as well as increased cell proliferation and migration, all contributing to PDAC development [89]. A dense fibrotic stroma also exerts mechanical stress (compression) on the tumor vessels and thus worsens tissue perfusion, restricts the oxygen/nutrient availability and induces hypoxia. Therefore, various therapeutic strategies have been designed to target HA in pancreatic solid tumors. Among the anti-stromal therapies, administration of hyaluronidase PEGPH20 to PDAC patients showed encouraging outcomes, but only in combination with chemotherapeutics, e.g., Gemcitabine or Nab-paclitaxel [89].

The interplay between cancer cells and the tumor milieu comprises the complex network of signaling cascades. Upregulation of Sonic (SHH), Indian (IHH) and Dessert (DHH) ligands of the Hedgehog signaling pathway is crucial for acinar cell healing after the episode of pancreatitis [90]. However, misregulation of the *SHH* expression was reported for the (pre)malignant pancreatic lesions PanINs, especially if these ligands were co-expressed with oncogenic *KRAS* [91]. High levels of SHH ligand contributes to development of desmoplastic reaction in pancreatic cancer, also by the induction of PSC activation [92]. To target the SHH signaling cascade in PDAC, a Hedgehog inhibitor IPI-269609 was tested in vitro and in vivo. Application of IPI-269609 affected the tumor growth; however in a combination therapy with Gemcitabine this inhibitor did not potentiate the effect of the chemotherapeutic [93]. Nevertheless, a newer-class inhibitor IPI-926 improved the delivery of drugs to cancer cells via normalization of the vascular network in the tumor [94].

Src/FAK is yet another important signaling cascade characterized by significant changes during tumorigenesis. Src and FAK may become activated by a number of growth factor receptors, mainly epidermal (EGFR), vascular endothelial (VEGFR), fibroblast (FGFR) and plateletderived growth factor receptor (PDGFR); all of the above could activate the Src/FAK pathway [95,96,97]. In PDAC, Src kinase activity is enhanced, which translates into increased cancer invasion and metastasis formation [98]. Src also becomes upregulated in pancreatitis a disease that substantially increases the risk of PDAC development [99]. Pharmacological inhibition of Src reduced cancer invasiveness and attenuated tumor growth [100,101], whereas blockage of FAK with its inhibitor VS-4718 (clinical trials) decreased the density of the TME and enabled the effective chemo- and immunotherapy [102].

## 4. Microenvironment: Cellular Components

### 4.1. Cancer-Associated Fibroblasts

Cancer-associated fibroblasts (CAFs) are abundant and heterogeneous populations of stromal cells in the microenvironment of pancreatic cancer, that are found both in the tumor itself as well as in its surroundings. CAFs can originate from a variety of cells: (i) activated tissue fibroblasts, (ii) transdifferentiated epithelial cells or pericytes, (iii) mesenchymal progenitor cells recruited into the tumor, and (iv) stem cells [103,104,105,106]. It has recently been shown that even pluripotent adipose tissue-derived stromal cells (ASCs) can be recruited from the peripancreatic areas into the tumor [107] and, in response to pro-tumorigenic signals, converted into CAFs [107].

Fibroblasts are spindle-shaped adherent cells involved in ECM protein turnover. They produce and release various cytokines or growth factors as part of the role they play in the regulation of tissue homeostasis. However, fibroblasts can also support tumor growth and cancer progression, promote metastasis formation and modulate tumor response to therapy [106,108]. Interestingly, CAFs may assume different phenotypes in distinct cancers/cancer compartments [104,109]. As such, based on their biology and function, CAFs can be divided into five populations: the tumor-promoting (*p*CAF), tumor-retarding (*r*CAF), secretory (*s*CAF), inflammatory (*i*CAF) and myofibroblast (*myo*CAF) [106]. Out of the above, *myo*CAFs and *i*CAFs are the most common and extensively studied CAF subpopulations in PDAC [105,110,111]. These cells are identified by expression of certain markers. For example typical stromal fibroblasts express alpha-smooth muscle actin (αSMA), chondroitin sulfate proteoglycan (NG2), fibroblast-activation protein (FAP) and fibroblasts specific protein-1 (FSP-1), as well as platelet-derived growth factor receptor (PDGFR) [104,105]. While αSMA and FAP are accepted to be the most specific markers for *myo*CAFs, *i*CAFs are characterized by particularly high expression of proinflammatory interleukin-6 (IL-6) [112].

Under normal conditions, local fibroblasts are quiescent cells with a limited ability to proliferate and migrate. However, a number of signals can promote fibroblast activation and differentiation into myofibroblasts; this includes TGFβ, SHH signaling, changes in the extracellular matrix stiffness, DNA damage, cell-to-cell or cell-to-fiber contact signals, and physiological stress [110]. In PDAC, PSCs are the most likely precursors of CAFs. In the healthy pancreas quiescent PSCs comprise approximately 4–7% of the organ mass and store vitamin A in cytosolic droplets [113,114]. Upon activation, PSCs lose vitamin A and start expressing αSMA [115,116,117]. However, their transition into *i*CAFs or *myo*CAFs depends on TGFβ and IL-1 signaling [118]. For instance, a very recent study by *Somerville* et al. has highlighted a role of p63 in the induction of the IL-1α-driven transition of PSCs into *i*CAFs in the squamous subtype of PDAC [119].

Once transdifferentiated into myofibroblasts, activated PSCs become involved in the production of ECM components, mainly collagens, fibronectin and laminin. This contributes to the tumor desmoplastic reaction one of the main hallmarks of PDAC. Clearly linked to the PSC activity, desmoplastic reaction in PDAC has been recognized as the main factor that worsens the outcome of anti-cancer therapies. In PDAC, dense desmoplasia severely limits vascular perfusion, as well as it decreases the nutrient availability and drug delivery to cancer cells [103]. In addition, CAFs are involved in cancer spread as evidenced by their presence in distant metastases [120].

Our understanding of the CAF influence on carcinogenesis and the efficacy of the therapy has increased over the years owing to the intensified research in the field. While CAFs can act as both the pro- and anti-tumorigenic effectors, their activation (for example - an increase in αSMA expression) and high abundance in the tumor tissue, are usually the indicators of a poor clinical outcome [121,122,123]. As demonstrated for a metastatic breast cancer, the presence of CAFs circulating in the blood could serve as an additional biomarker of disease severity [124]. Also, a growing body of evidence demonstrates that CAFs contribute toward drug resistance via different mechanisms, including paracrine signaling (e.g., secretion of IL-6, IGF1 or NO, and SDF-1/SATB-1 pathway), or by formation of a dense physical barrier (the stroma) that affects drug delivery to cancer cells [106,108].

Novel therapeutic approaches target mainly those signaling pathways that lead to CAF activation (e.g., TGFβ signaling), or that aim to restore fibroblast quiescence. Alternative approaches based on complete depletion of CAFs from PDAC unexpectedly resulted in the increased aggressiveness of the tumor [125]. Even selective targeting of the αSMA-positive CAFs in the mouse model of PDAC induced immunosuppression and accelerated progression of the disease [126].

These examples highlight the importance of further studies on the role(s) of CAFs in the development and progression of PDAC. However, the lack of highly-specific CAF markers, a significant heterogeneity of CAF populations and unclear influence of CAFs on the pancreatic carcinogenesis make the future studies particularly challenging [127].

### 4.2. Immune Environment in PDAC

Chemotherapy, often in combination with surgery or radiotherapy, still remains the major therapeutic intervention against pancreatic cancer. However, a growing body of evidence suggests that immunotherapy could improve the outcome of these interventions [128]. While tailoring new therapeutic strategies it pays to remember that solid tumors contain populations of anti- and pro-tumor immune cells that could modulate the outcome of the therapy [129,130]. Indeed, in recent years this fact is becoming increasingly more acknowledged in the treatment of solid tumors, including PDAC [131].

#### 4.2.1. Immune Checkpoint PD-1/PD-L1

Programmed cell death 1 (PD-1; also known as CD279) is an inhibitory checkpoint molecule that regulates self-tolerance toward different cell types and protects against the immune attack by cytotoxic T cells and pre-B cells [132]. However, when expressed in tumor, this protein prevents the immune system from killing neoplastic cells. It is now commonly accepted that blockade of immune checkpoints in certain cancers may be beneficial in cancer therapy [133]. Nevertheless, as evidenced by a recent analysis of the clinical data collected up to November 2018, checkpoint inhibition monotherapy has so far proven ineffective in pancreatic cancer [134].

The presence of local immune response in the tumor is often used as a prognostic factor of the clinical outcome. It was demonstrated that a high number of tumor-infiltrating lymphocytes (TILs) in the stroma provided a reliable indicator for the overall survival (OS) of PDAC patients as well as a predictor for the absence of liver metastases [135]. In one study, 145 pancreatic cancer patients who had undergone pancreatectomy followed by postoperative chemotherapy (mostly Gemcitabine) were tested for TIL number and their distribution in relation to expression of PD-1, its ligand (PD-L1; expressed on macrophages, epithelial and other normal cells), as well as CD8 and FOXP3 in tumor-infiltrating leukocytes [135]. A multivariable analysis demonstrated that PD-1 and CD8, but not PD-L1 and FPXP3, when expressed in TILs, could be associated with better OS as well as local progression- and distant metastases-free survival (LPFS and DMFS, respectively) [135]. This is in line with another study, which suggests that lower PD-L1 expression in the surgically resected tissues, may be a good prognostic factor for the patients independently on the level of TIL [136].

In developing tumors, not only immune cells, but also cancer cells express PD-1. A very recent preclinical study applied RNA-Seq to examine the expression of *PD-1* gene in the library of hundreds of human cancer cell lines [137]. As a result, pancreatic cancer cell lines (*n* = 46) exhibited higher PD-1 expression than most solid tumor cell lines, even those of other different gastrointestinal cancers [137]. This study also shows that PD-1 expression enhances PDAC growth: PANC-1 cells with stably transfected PD-1 shRNA xenotransplanted into NOD/SCID mouse hosts grown much smaller tumors (~67% lower volume) compared to scrambled shRNA control tumors [137]. The authors of this study also highlighted the importance of PD-1/PD-L1 interaction in activation of mitogen activated-protein kinase (MAPK) signaling and thus regulation of cell proliferation and survival [137].

#### 4.2.2. Current Strategies to Modulate Immune Checkpoint Blockade (ICB)

Since attempts to block PD-1/PD-L1 in pancreatic cancer have proven largely disappointing, a number of studies tested combination therapies to target the immune checkpoints in PDAC. To address the problem of poor effectiveness of PD-L1 inhibitors applied as monotherapy, a preclinical study examined the interactome of PD-L1 [138]. This study explored the GEPIA database [139] and found that histone acetyltransferase 1 (HAT-1), a transcriptional regulator of PD-L1, was upregulated in PDAC and associated with the poor prognosis [138]. The authors showed that knocking-down the *HAT1* gene decreased PD-L1 and improved the efficacy of immune checkpoint blockade (ICB) [138]. Another preclinical study carried out in the MUC1.Tg mice examined the possibility of immunization of the tumor host with mAb-AR20.5 a tumor antigen-targeting antibody (here: anti-mucin 1, MUC1) [140]. This procedure was then followed by administration of PolyICLC (to induce interferon system in mice [141]) together with anti-PD-L1. This combination resulted in a specific, long-lasting, adaptive response against Panc02.MUC1 tumors. As a result, this study proposed a proof-of-principle strategy of targeting a tumor specific antigen, which, if coupled with agents that act as a vaccine adjuvant and an immune checkpoint inhibitor, could provide an effective and long lasting immunity against PDAC [140]. ICB in a combination with radiotherapy was tested in vitro and in vivo against human and mouse pancreatic cancer models [142]. Radiation not only improved ICB efficacy, but also induced DNA damage, which is a well-known activator of the innate immune response [143]. Radiation-induced signaling pathways, including ATM serine/threonine kinase (ATM) signaling, was modified with a pharmacological ATM inhibitor KU60019 as well as it was blocked genetically [142]. Of note is that the ATM-deficient cancer cells formed larger tumors in the immunosuppressive mouse hosts (NSG; they lack the T, C and NK cells) compared to the syngeneic immunocompetent (FVB/NJ or C57BL/6) hosts [142]. In the latter hosts, the growth of the tumors with genetically silenced expression of the *ATM* gene (shATM) or the scrambled control (shCon) tumors was monitored in response to anti-PD-L1/radiation or combination of those. The approach that combined anti-PD-L1 with radiation either reduced or even abolished the growth of shATM tumors [142]. The authors investigated the mechanisms of down-stream ATM signaling pathway in vivo and showed upregulation in the interferon response. In conclusion, the authors of this study suggested that ATM inhibition induces interferon-mediated innate immunity against PDAC, and by increasing the sensitivity to anti-PD-L1 therapy, may prove beneficial for the therapy outcome if combined with radiotherapy [142]. T-cell checkpoint immunotherapy in PDAC was also investigated in the context of tumor-associated macrophages (TAMs) and their immunosuppressive effect on the therapy outcome [144]. By blocking colony stimulating factor/its receptor CSF1/CSFR1, a depletion of macrophages characterized by a high expression of mannose receptor (CD206) was achieved [144]. Depletion of CD206^high^ TAMs and reprogramming of the others, when combined with immune checkpoint blockade with PD-1 or cytotoxic T-lymphocyte-associated protein 4 (CTLA-4) antagonists, improved the anti-tumor immunity [144]. Another study investigated cytokine IL35, an immunosuppressive driver of PDAC growth, in relation to T cell populations in this tumor. A synergistic effect of IL35 deficiency, achieved by knocking down *Ebi3* gene, and anti-PD-1 treatment was reported [145].

#### 4.2.3. T Cells in Focus

T cell populations still remain relatively underexplored in the context of (pre)clinical pancreatic studies. To illustrate this, it was only in 2019 when a previously underappreciated population of unconventional T cells, TCRαβ^+^CD4^–^CD8^–^NK1.1^–^ innate αβ T-cells (iαβTs), was identified to represent as much as ~10% of T-lymphocytes in PDAC solid tumors (both in mouse and human) [146]. The most current ’trend’ in research on T-cells is centered on ex vivo biotechnological engineering of T-cell receptors so that the chimeric artificial receptors (CARs) are expressed on T-cell surface [147]. A novel cell class, the CAR-Ts, carry the genetically redirected receptors that combine antigen-binding and T-cell activating functions in a single receptor [147]. Originally proposed to treat blood malignancies (leukemias and lymphomas [147,148]), CAR-Ts were repurposed as agents against the solid tumors [148,149]. However, solid tumors are often very poorly-perfused and have a dense, fibrotic and highly immunosuppressive microenvironment, which makes the application of CAR-Ts technically challenging. Advances on CAR-T in PDAC treatment were review in recent articles [148,149,150].

## 5. Microenvironment: Stress and Stress-Induced Signaling

### 5.1. Hypoxia

A decreased level of oxygen supply to the tissue results in the development of hypoxia. This condition is characterized by a reduced oxygen level, inadequate to the local body needs. Interestingly, hypoxia is a hallmark of the solid tumors, including PDAC [151]. The first study to assess the extent of hypoxia in pancreatic carcinomas was published 20 years ago [152]. In this clinical investigation a group of seven patients gave their consent to have tumor oxygenation measured intraoperatively using a polarographic electrode [152]). The study revealed a marked difference in the oxygen partial pressure (pO_2_) measured within the tumor and the adjacent normal pancreatic tissues [152]. In this study, the pancreatic neoplasias were shown to be highly hypoxic, of median pO_2_ as low as 0.0–5.3 mmHg (1 mmHg = 0.13% O_2_, 133.3 Pa [153]). Only 6% of the tumor tissue was characterized by the median pO_2_ higher than 2.5 mmHg [152]; whereas in the normal pancreatic tissues surrounding the tumor, the median pO_2_ were much higher and reaching 24.3–92.7 mmHg.

Development of a hypoxic fraction in PDAC is influenced by a number of factors, for example a very rapid tumor growth may result in impaired architecture of the tumor vasculature. This largely affects O_2_ delivery to the tissue [154]. What is more, tumor microenvironment may decrease angiogenesis via the autocrine activity of PSCs, such as secretion of endostatin [154,155]. Also, the physical characteristics of TME, such as its very high density, exerts physical pressure that not only causes hypovascularization in the tumor, but also further limits the blood flow in the dysfunctional tumor vessels [155]. These reactions drive a vicious cycle that links desmoplasia with hypoxia. Hypoxia also increases expression of proteins involved in drug efflux and induces cancer cells stemness [151,156].

In order to survive in the limiting microenvironment they inhabit, cancer cells evolved a number of strategies to protect themselves despite the stress conditions. Hypoxia may throw cell homeostasis off balance and thus limit their capabilities to survive. Hypoxia-inducible factors (HIFs) are transcription factors that allow the maintenance of homeostasis under the low availability of O_2_ [157]. Stability of HIFs is controlled via post-translational modifications, such as hydroxylation and ubiquitination [151]. Constant hydroxylation of a HIF1α subunit makes HIF1 unstable under normoxic conditions. However, HIF1 becomes stabilized in hypoxia and translocates to the nucleus, where its subunits form heterodimers and bind to the hypoxia response elements (HREs). This regulates transcription of genes that encode proteins involved in angiogenesis (vascular endothelial growth factor VEGF, and its receptors; platelet-derived growth factor B - PDGF-B; heme oxygenase-1 - HO-1), metabolism (glucose transporters - GLUT1 and GLUT3, phosphoglycerate kinase 1 - PGK1), proliferation (cyclin D1; TGFβ) and invasion (stromal-derived factor 1 - SDF-1; E-cadherin) [158,159].

By exploiting their ability to switch from oxidative phosphorylation to glycolysis, HIFs promote cancer cell survival under hypoxic conditions [157]. Moreover, hypoxia-stabilized HIFs can contribute to EMT of the pancreatic epithelia into malignant neoplastic cells characterized by a high metastatic potential [160]. Reduced O_2_ levels also facilitate stromal remodeling, further supporting cancer invasion [160]. Finally, hypoxia signaling modifies the interactions between cancer cells and non-neoplastic cells in PDAC: CAFs, PSCs and macrophages, and other cells [160]. What is more, by increasing the expression of granulocyte-macrophage colony-stimulating factor (GM-CSF), HIF1α promotes Schwann cell migration, perineural invasion and hypersensitivity to pain-inducing stimuli in the PDAC patients [161]. Not surprisingly, expression of HIF1α is also associated with a bigger tumor size, worse prognosis and increased PDAC invasiveness and metastatic potential [160,162,163,164]. Several studies confirmed that HIF expression levels can be used as a predictor of the therapy outcome [157]. A growing body of research reveals a correlation between the extent of the hypoxic regions within the tumor and a decreased efficacy of the therapy, particularly radiotherapy [152]. Therefore, assessment of the hypoxic fraction in pancreatic solid tumor prior to the treatment could markedly improve the therapy outcome. Radiotracer fluoromisonidazole [^18^F] FMISO was shown to be effective in hypoxia imaging in PDAC: its tumor to background ratio was 2.0, and it allowed for 120–150 min imaging time [154].

The main hypoxia response regulators HIFs are the potential targets for anticancer therapies, either *per se* or in an adjuvant fashion. Various approaches to target HIF1α have already been undertaken. EZN-2968, an antisense oligonucleotide against HIF1α mRNA was tested in a pilot clinical trial [165]. However, despite a decreased level of HIF1α protein, expression of its target genes was not significantly reduced [165]. Minnelide, a drug used to inhibit the transcriptional activity of HIF1α and decrease the level of pancreatic cancer cell stemness [166], has been evaluated against PDAC in phase II clinical trials [167]. The inhibition of downstream HIF effectors, e.g., HO-1, is another plausible therapeutic strategy to target hypoxia. A very recent study revealed that HO-1 knockdown in human PDAC cell lines resulted in a significant inhibition of cell proliferation under hypoxic conditions [162]. Moreover, as studied in PDAC xenograft tumors grown in a mouse host, HO-1 inhibition combined with Gemcitabine treatment markedly increased the efficacy of this chemotherapeutic [162].

For improving therapy effectiveness, also blockade of signals that lead to HIF upregulation could prove beneficial [157]. In principle, inhibition of the mTOR signaling pathway that controls HIF1α translation could be a promising therapeutic approach for cancer therapy. Disappointingly, no significant clinical activity was demonstrated using the mTOR inhibitor Everolimus as a monotherapy to treat Gemcitabine-refractory metastatic pancreatic cancer [168]. However, after several years of unsuccessful attempts, Everolimus again was proposed for clinical tests in combination with other anticancer drugs [169].

A relatively novel group of compounds called hypoxia activated prodrugs (HAPs) were designed to specifically target hypoxic environment and thereby improve anticancer effectiveness. The HAPs, often derived from 2-nitroimidazoles, comprise cytostatic and cytotoxic compounds [170]. Under hypoxic conditions, HAPs undergo redox reactions in which they yield chemical agents that decrease cell proliferation or cause cancer cell death. Probably the most recognizable prodrug of this class, TH-302 (also known as Evofosfamide), was used unsuccessfully in a number of clinical trials on different types of cancer, including PDAC [171]. However, one clinical trial (NCT03098160) on immunotherapy combining Ipilimumab and Evofosfamide to target metastatic pancreatic cancer, is still active, but its efficacy is yet to be assessed.

### 5.2. Microbiome

Intricate relation between the microbiome and carcinogenesis is gaining a lot of interest in the field of cancer-related research. Analysis of microflora revealed particular microbe species that inhabit patient-derived PDAC solid tumors, as well as the relation of these species to gut microbiome. *Gammaproteobacteria* are the most abundant bacteria class that colonizes the tumors [172,173]. Interestingly, this particular microbe expresses bacterial enzyme cytidine deaminase (CDDL) that can convert a chemotherapeutic Gemcitabine into its inactive form, 2′,2′-difluorodeoxyuridine [172]. Hence, microbiome may have a direct impact on PDAC chemoresistance. In a mouse model of pancreatic cancer, deprivation of bacteria via oral antibiotic administration was shown to induce remodeling of TME, shifting it from an immunosuppressive state, promoting T-cell activation and differentiation of macrophages in the tumor. This antibacterial treatment also enhanced PD-1 expression, further improving tumor sensitivity to immunotherapy [174]. Although in colon and gastric cancers pathogenic oncogenesis is a direct pathway for disease development, in pancreatic cancer bacterial influence seems of rather indirect nature. As such, it is in conjunction with the oncogenic KRAS when the presence of pathogens throws the Th1/Th2/Th17 cell ratio off balance and accelerates cancer progression [175].

Another plausible pathway of the microbiome-PDAC crosstalk depends on the pancreatic cancer risk factors, such as pancreatitis or diabetes. These often severe conditions cause microbial dysbiosis and increase the gut permeability, further promoting translocation of gut microbes to the pancreas. As a result, Toll-like receptors (TLRs) become activated in the pancreatic parenchyma by the bacterial ligands, what expedites cancer growth [176]. However, consensus on pro- or anti-tumorigenic influence of microbiome has not yet been reached. For example, in contrast to the aforementioned reports some recent studies indicate that high alpha diversity (a parameter that takes into account species richness, evenness/domination) of the microflora is tightly correlated with a good OS in PDAC patients, whereas a low level of alpha diversity may serve as a prognostic marker for a poorer disease outcome. Based on these data, in the early-stages of PDAC progression, composition of patient oral and gut microbiome could be a prognostic factor for estimating the survival success rate [176,177].

Evidence suggests that oral microbial ecosystem, likewise, contribute toward the diversification in prognosis of PDAC patients. Salivary levels of *Neisseria elongata* and *Streptococcus mitis* were markedly lowered in patients with pancreatic cancer compared to the control group [178]. On the contrary, *Granulicatella adiacens* content increased in PDAC cases, which may suggest its correlation with systemic inflammation [178]. Also, the transfer (via contact) of periodontal-associated bacteria *Porphyromonas gingivalis* and *Aggregatibacter actinomycetemcomitans* could be related to the higher risk of pancreatic cancer [179]. Hence, changes in oral microbial content may serve as a non-invasive and inexpensive screening tool for the patients.

### 5.3. Altered Metabolism

From the survival standpoint, metabolic adaptability is a crucial feature of cells exposed to the dynamic environmental conditions [180]. Such pressure may pose a challenge even for cancer cells. This is particularly true for pancreatic cancer, since the dense fibrotic stroma in PDAC forms a physical barrier for effective nutrient and oxygen delivery. Therefore, in order to survive, pancreatic cancer cells must develop mechanisms altering the typical cell metabolic pathways and thus supply the essential nutrients. Such metabolic rewriting in cancers often implies the prevalence of the anaerobic glycolysis [181] and dependence of cancer on the glycolytic pathway [182]. A phenomenon known as the Warburg effect - more common in cells that exist in the limiting environment, such as cancer cells - is based on a shift from glucose oxidation to other anabolic pathways, such as pentose phosphate pathway (PPP) [183].

Uptake of nutrients exceeding the typical cell needs and increased cell proliferation are facilitated by various oncogenic mutations, including *KRAS* point mutations and missense *TP53*. Oncogenic KRAS regulates glucose metabolism through upregulation of the glucose transporter GLUT1 and glucose-transforming enzymes hexokinases (HEK) as well as phosphofructokinase 1 (PFK1) [184]. Oncogenic KRAS also markedly affects the glutamine pathway, which in cancer cells appears to play a pivotal role in nutrient dispensing [185]. Apart from conversion of glutamate to α-ketoglutarate using glutamate dehydrogenase 1 (GLUD1), cancer cells utilize glutamic-oxaloacetic transaminase 1 (GOT1) to generate oxaloacetate from glutamine-derived aspartate. Oxaloacetate is then converted into malate and subsequently pyruvate that fuels tricarboxylic acid (TCA) cycle [185]. *KRAS* mutation, resulting in a decrease of GLUD1 with a simultaneous increase in GOT1, reprograms metabolism in favor of the glutamine pathway. Importantly, the influence of the oncogenic *TP53* synergizes with mutated *KRAS* in cancer metabolic alterations [186]. As illustrated by a recent study, when p53^R270H^ mutation is accompanied by KRAS^G12D^, they both slow down the branched-chain amino acid (BCAA) metabolism in cancer cells; this may explain the reduction in mitochondrial activity [187]. Another means for targeting TCA in cancer cells is a pharmacological blockade of α-ketoglutarate dehydrogenase complex with its inhibitor CPI-613 [187]. This strategy, in combination with FOLFIRINOX, showed promising results in metastatic pancreatic cancer cases [187].

Of note, since aldohexose mannose is similar to glucose, it could interfere with glucose metabolism and modify the levels of the TCA intermediates, important for cancer growth. Mannose supplementation was also tested in combination with cisplatin chemotherapy. Importantly, cisplatin/mannose treatment amplified apoptosis in cancer cells by down-streaming the anti-apoptotic Bcl-2 proteins [188].

Not only glucose and glutamine fuel TCA cycle. Activated PSCs support cancer metabolism through secretion of a non-essential amino acid (NEAA) alanine [189]. This phenomenon is tightly related to autophagy, since it was evidenced that non-autophagic PSCs do not ‘feed’ cancer cells with NEAAs [189]. The role of autophagy turned out to be critical in regulation of cancer cell proliferation (here: by switching cell metabolism toward anaerobic) and metastasis formation [190]. As such, autophagy repression opens up a therapeutic avenue for pancreatic cancer treatment, also in combination with other approaches [182,190].

Lipid metabolism is a rapidly expanding research area, also in relation to pathophysiology. Since pancreatic cancer cells utilize lipids and fatty acids as a source of energy, it becomes of increasing interest to understand the role these nutrients play in the dysregulated PDAC metabolism [191]. Up-regulation of lipoprotein and cholesterol pathways in PDAC captured scientific attention as potential targets for novel therapeutic strategies. In one study, expression of the low-density lipoprotein receptor (LDLR) was used for cholesterol depletion in a combination therapy, which has proven more effective than Gemcitabine alone [191].

High-fat diet enhances pancreatic cancer progression through elevation in cholecystokinin (CCK) levels [192]. Furthermore, as studied in a KPC mouse model of pancreatic carcinogenesis, high-fat diet resulted in increased size of primary tumors and the higher rate of distant metastasis compared to the control KPC mice fed on a normal-fat diet [193]. In PDAC, pro-tumorigenic effect of lipids on disease progression also relates to the processes of PSC transition into CAFs. Remodeling of PSC lipidome and lipid secretion was shown to support tumor growth by supplying cancer cells with phosphatidylcholines for membrane synthesis, as well as increasing lysophosphatidic acid (LPA) production. Since the latter plays a role in wound-healing, it may also support the processes of desmoplastic reaction in PDAC and progression of the disease [194].

### 5.4. High Fat Diet, CCK Receptors and PDAC

Genetic susceptibility to cancer is very well recognized and firmly rooted in the public awareness. Course-book examples include certain mutations in the *BRCA1* and *BRCA2* genes that significantly increase the risk of developing breast and ovarian cancers [195]. However, environmental factors, such as our diet, also play an important role in carcinogenesis. Substantial evidence has accumulated in recent years and points toward a fatty acid-rich diet as a significant risk factor of pancreatic cancer [196,197]. In contrast, low-fat diets are not associated with an increased incidence of the disease [198]; and omega-3 fatty acids may even be able to decrease the risk [199].

Fatty acids are important modulators of CCK secretion by the I-cells of the duodenum [200], a hormone and one of the most important secretagogue for digestive enzymes released by PACs [201]. What is more, it has long been known that CCK at physiological concentrations induces hypertrophy (increase in organ mass) and hyperplasia (increase in cell number) of the pancreas [202,203]. While CCK-A receptor is expressed in the normal mouse pancreas and CCK-B receptor is present in the normal human tissue [204], both CCK-A and CCK-B receptors have been identified on human pancreatic cancer cells as well as in PanINs from mouse and human tissues [205,206,207]. Since the above indicates that CCK receptors are important for pancreatic cancer development, they might be a viable target for therapeutic intervention. In support of this claim *Smith* et al. showed that inhibition of both CCK receptors with Proglumide delays the progression of PanINs in mice as well as reduces fibrosis and inflammation in vivo [207]. CCK receptor inhibition was also demonstrated to enhance the response to immunotherapy in pancreatic cancer: simultaneous blockade of CCK receptors and application of immune checkpoint antibody, PD-1 or CTLA-4, significantly decreased the tumor volume, improved survival of the experimental mice and had an effect on the type and number of immune cells found in the tumor microenvironment [208].

In line with the above reports, it has recently been shown that the same CCK receptor antagonist blocks dietary fat-induced growth of pancreatic cancer cells, inhibits metastases in vivo as well as decreases tumor fibrosis; whereas CCK analogue is able to stimulate the growth of Panc02 cells in vitro as long as they possess a functional CCK receptor [192]. Importantly, CCK-KO mice or pancreatic tumors that lack CCK receptors are not affected by dietary fat [192]. Reducing the intake of fat could be an important factor in lowering the risk of pancreatic cancer.

### 5.5. Ca^2+^ Signaling in PDAC

Ca^2+^ ions are possibly the most versatile signaling entities that control a wide spectrum of cellular processes, including cell migration, proliferation as well as cell death. Given that these processes are crucial for carcinogenesis, the role of Ca^2+^ signaling in cancer is widely accepted and has been a subject of numerous reviews in the past [209,210]. An increasing number of studies provide evidence for the role of Ca^2+^ and Ca^2+^ permeable channels in the development of different cancer types [211,212]. Ca^2+^ signaling clearly plays a role in the tumor growth as well as affects its invasive and metastatic properties [213]. However, only very recently we have begun to understand the role of Ca^2+^ channels specifically in pancreatic tumorigenesis.

Store operated Ca^2+^ entry (SOCE) is the most important Ca^2+^ influx pathway in non-excitable cells and the major mechanism that replenishes the ER Ca^2+^ store [214]. This pathway is triggered in response to a depletion of the intracellular Ca^2+^ stores and it is mediated by the interaction of two molecules: a plasma membrane channel Orai1 and a Ca^2+^ sensor stromal interaction molecule 1 (STIM1) located in the ER [215]. Orai1 and STIM1 not only facilitate SOCE in non-transformed cells but also function in different PDAC cell lines playing pro-survival and anti-apoptotic roles [216]. Both STIM1 and Orai1 have previously been shown to play a role in various types of malignancies, such a cervical cancer, breast cancer or prostate cancer [217,218,219]. Very recently *Wang* et al. demonstrated that STIM1 expression becomes upregulated in human PDAC tissues and the authors suggested that this protein might be a novel predictive and prognostic marker for pancreatic cancer [220]. Expression of STIM1 showed a very similar pattern to HIF1α both in PDAC cell lines and human samples and it was postulated that the STIM1 promoter activity is regulated under hypoxic conditions by HIF1α via its binding to HREs on this promoter [220]. What should be taken into consideration is the fact that both Gemcitabine and 5-fluorouracil might be able to upregulate Orai1 and STIM1 even further, increasing SOCE in PDAC and thus making the disease even more resilient to chemotherapy [216]. A very recent report showed that RP4010, a SOCE blocker, inhibited the proliferation of pancreatic cancer cell lines and pancreatic tumor-derived cells; as well as demonstrated that the anti-tumor properties of this pharmacological agent were enhanced in the presence of standard chemotherapy with Gemcitabine or Nab-paclitaxel [221].

Another target, whose importance has been highlighted by recent studies, is P2X7, an ion channel activated by ATP and permeable for Ca^2+^. Its expression was found in pancreatic cell lines, primary tumor cells as well as mouse PSCs [222,223]. While high concentrations of exogenously applied ATP (as well as P2X7 receptor agonist, BzATP) caused pore formation followed by cell necrosis in PDAC cell lines, inhibition of this receptor with AZ10606120 slowed down cell proliferation [222]. Importantly, this compound also decreased the tumor growth (measured by bioluminescence in vivo) in an orthotopic xenograft mouse model of PDAC, reduced the number of activated PSCs (seen as decreased α-SMA expression) as well as inhibited collagen deposition [223]. The authors hypothesize that P2X7 receptor plays a role in PDAC progression and thus its inhibition might ameliorate pancreatic cancer aggressiveness by affecting PDAC proliferation, interaction between cancer cells and PSCs and by reducing the desmoplastic reaction.

## 6. Conclusions

The term ‘ecosystem’ has been used for decades to address the intricacies of pancreatic solid tumor and acknowledge the complexity of PDAC. Not only it well reflects the abundance of cell types that build the tumor, but also it highlights the dynamics of the cell-cell and cell-matrix interactions.

Although we have accumulated a substantial knowledge about the mechanisms that drive pancreatic carcinogenesis and there have been proposed multiple avenues of therapeutic intervention, PDAC remains a global health problem and a major burden to the financial system. Therefore, not only more research is required to harness the mechanisms that underlie the development of this deadly disease, but also more complex, multidisciplinary treatment approaches are needed.

Better understanding of the biophysical processes present in the acellular component of the stroma may contribute toward the more insightful characteristic of the cells that inhabit tumor microenvironment. In light of recent findings, not only the body’s own eukaryotic cells, but also the prokaryotic microbiome, are the vital players in PDAC development and plausible therapeutic targets in PDAC therapy.
